# Environmental Exposures and Lung Aging: Molecular Mechanisms and Implications for Improving Respiratory Health

**DOI:** 10.1007/s40572-021-00328-2

**Published:** 2021-11-04

**Authors:** Christina M. Eckhardt, Haotian Wu

**Affiliations:** 1 Department of Medicine, Division of Pulmonary, Allergy and Critical Care Medicine, Columbia University Irving Medical Center, 630 West 168th Street, Presbyterian Hospital Floor 8, Suite 101, New York, NY 10032 USA; 2 Environmental Health Sciences Department, Columbia University Mailman School of Public Health, 630 West 168th Street, Room 16-416, New York, NY 10032 USA

**Keywords:** Environmental exposures, Air pollution, Smoking, Occupational exposures, Lung aging, Lung disease

## Abstract

**Purpose of Review:**

Inhaled environmental exposures cause over 12 million deaths per year worldwide. Despite localized efforts to reduce environmental exposures, tobacco smoking and air pollution remain the urgent public health challenges that are contributing to the growing prevalence of respiratory diseases. The purpose of this review is to describe the mechanisms through which inhaled environmental exposures accelerate lung aging and cause overt lung disease.

**Recent Findings:**

Environmental exposures related to fossil fuel and tobacco combustion and occupational exposures related to silica and coal mining generate oxidative stress and inflammation in the lungs. Sustained oxidative stress causes DNA damage, epigenetic instability, mitochondrial dysfunction, and cell cycle arrest in key progenitor cells in the lung. As a result, critical repair mechanisms are impaired, leading to premature destruction of the lung parenchyma.

**Summary:**

Inhaled environmental exposures accelerate lung aging by injuring the lungs and damaging the cells responsible for wound healing. Interventions that minimize exposure to noxious antigens are critical to improve lung health, and novel research is required to expand our knowledge of therapies that may slow or prevent premature lung aging.

## Introduction

The human lung is continuously exposed to inhaled agents and pathogens from the external environment. A combination of individual genetics and environmental exposures influence lung aging, which manifests as structural remodeling of the respiratory tract that generates declining lung function over time [1]. During normal breathing, the trachea conducts air through the bronchi, which divide into bronchioles and end in clusters of alveoli. The alveoli are lined by an epithelial layer and basement membrane that lay adjacent to a thin interstitial space, under which lies the pulmonary capillary network [2]. The interstitial space contains lung extracellular matrix, which consists of elastic and collagen fibers that maintain the structural integrity of the lung [3]. Age-related weakening of the connective tissue in the lung generates progressive dilation of the airspaces and early collapse of the small airways [4]. The surface area of lung available for gas exchange thereby decreases with age, leading to reduced oxygenation and capacity for exercise [5]. Structural changes are even more pronounced in age-related respiratory diseases including chronic obstructive pulmonary disease (COPD), which is characterized by mucus hypersecretion and alveolar wall destruction, [6] and idiopathic pulmonary fibrosis (IPF), which is defined by interstitial fibrosis [7].

Age-related structural alterations in the lung are driven in part by inhaled exposures that damage the lung epithelium and underlying tissue [8]. Efficient repair mechanisms are critical to maintain the structural integrity of the lung and prevent pathological remodeling. Specialized type II alveolar epithelial cells contribute to wound healing by generating new type I epithelial cells after injury, which cover most of the alveolar surface [9]. Wound healing is also stimulated by multipotent mesenchymal stem cells that populate subepithelial lung tissue and differentiate into epithelial cells, macrophages, and reparative fibroblasts [10, 11]. Pulmonary fibroblasts reside in the interstitial space and mend the lung extracellular matrix in order to re-establish and maintain alveolar architecture [3]. However, when lung progenitor cells and cellular repair mechanisms are inhibited, aberrant structural remodeling distorts the lung architecture and leads to premature lung function impairment [1].

**Fig. 1:**
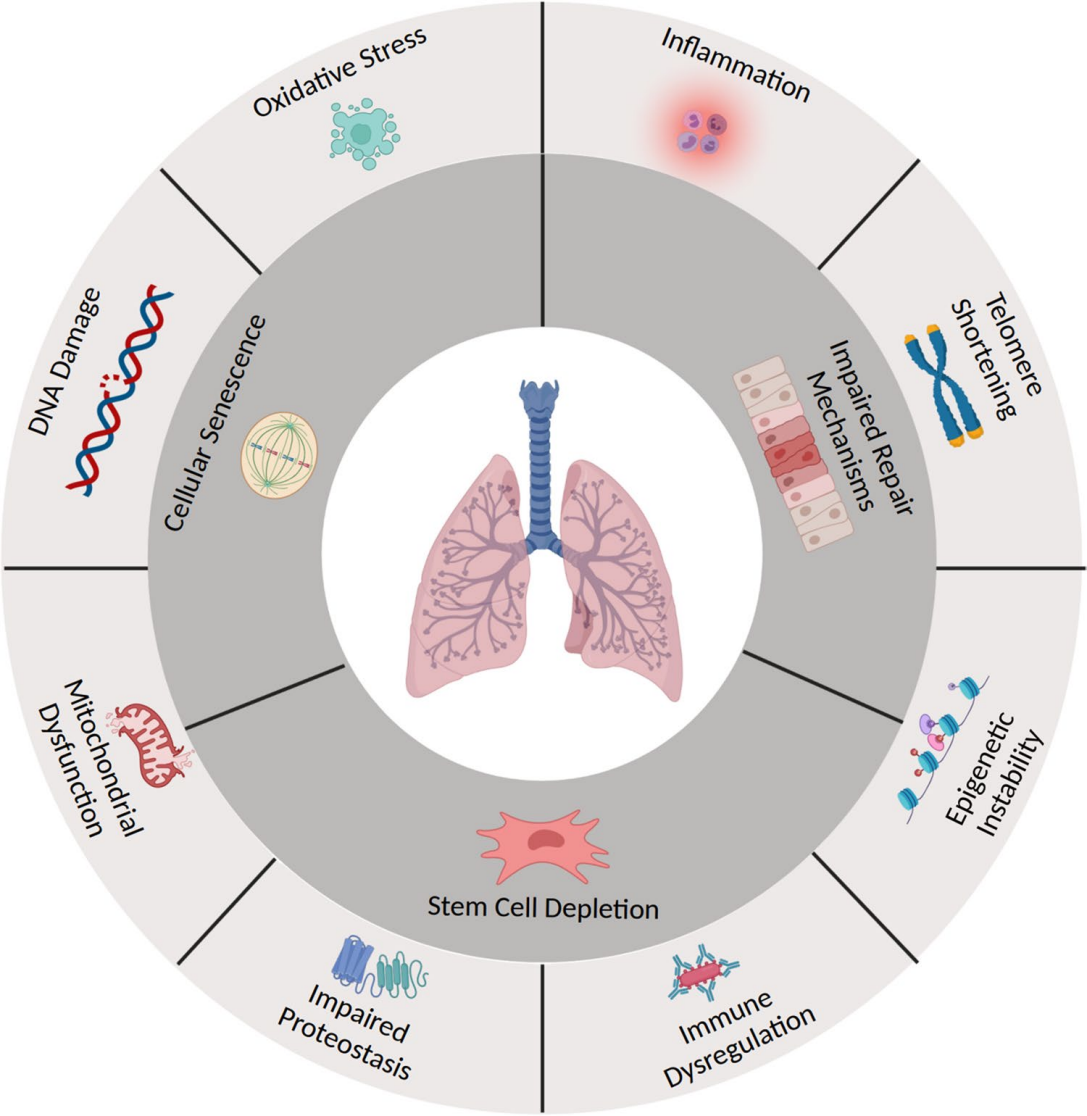
Mechanisms of lung aging induced by environmental exposures^141^. Figure 1 enumerates the biological pathways through which environmental exposures accelerate lung aging. Inhalation of noxious antigens induces oxidative stress, inflammation, telomere shortening, DNA damage, mitochondrial dysfunction, epigenetic instability, immune dysregulation, and impaired proteostasis in multiple cell lines in the lung. As a result, pulmonary stem cells are depleted, key progenitor cells become senescent, and critical repair mechanisms are impaired, leading to premature lung aging

Compromised cellular repair mechanisms are one of the hallmarks of lung aging (Figure 1). Inhaled exposures diminish the lung’s regenerative potential by generating oxidative stress, DNA damage, epigenetic instability, telomere attrition, mitochondrial injury, and abnormal protein homeostasis in key progenitor and structural cells [12••]. Accumulated damage in mesenchymal stem cells leads to apoptosis and stem cell depletion, while repeated insults in type II alveolar epithelial cells and lung fibroblasts lead to cellular senescence [13]. Cellular senescence is characterized by arrested growth and diminished cellular function, and cell-specific senescence can generate different forms of lung degeneration [14]. For example, senescent alveolar epithelial cells are unable to induce re-epithelialization after lung injury, while senescent fibroblasts produce aberrant collagen in the lung extracellular matrix [15, 16]. Cumulative inhalational exposures over the lifespan introduce accumulating inflammatory-oxidative stress and act in concert to induce widespread pulmonary cellular senescence and premature lung aging [17, 18]. This review provides an overview of environmental exposures that can impact lung health and details the mechanisms and biological pathways through which environmental exposures accelerate lung aging.

## Environmental Exposures and Biological Impact on the Lung

### Tobacco Smoke

Tobacco smoking is a combustion process that leads to the aerosolization of thousands of toxic chemicals including carbon monoxide, hydrogen cyanide, and polycyclic aromatic hydrocarbons [19]. Many of the components in tobacco smoke chemically react with oxygen to generate free radicals and inhibit protective antioxidants [20]. Through the combustion of noxious chemicals and generation of harmful reactive oxygen species (ROS), tobacco smoke induces widespread tissue damage in a manner that mimics biological aging (Table 1) [21].

**Table 1:** Summary of smoking-induced changes that accelerate lung aging

Reference	Age-related change	Summary	Physiologic effect associated with age-related change
Joehanes et al. [22]	Epigenetic instability	Tobacco smoking alters DNA methylation levels at thousands of CpG sites that regulate expression of over 7000 genes	Smoking-induced changes in DNA methylation alter expression of genes implicated in premature emphysema [27••]
Bradley et al. [42]	Abnormal proteostasis	Tobacco smoking precipitates impaired protein folding in the endoplasmic reticulum of lung cells	Accumulation of dysfunctional proteins inhibits surfactant protein production and triggers alveolar wall destruction [44]
Walters et al. [49]	Telomere shortening	Tobacco smoking reduces telomere length in lung epithelial cells, basal progenitor cells, and fibroblasts, leading to cell cycle arrest	Disordered cell differentiation and impaired epithelial remodeling generate functional and architectural disturbances in the alveoli and airways [50]
Goldfarbmuren et al. [54••]	Inflammation	Persistent smoking-related inflammation induces an airway epithelial-mesenchymal transition	Transformed mesenchymal cells produce a disorganized extracellular matrix and generate pulmonary fibrosis [53]
Bhat et al. [59•]	Immune dysregulation	Tobacco smoking suppresses macrophage phagocytosis of bacteria and inhibits B and T cell immune responses	Dysregulated immunity leads to repeated and prolonged respiratory tract infections that can induce structural changes in the lung [60]

Abbreviations: *CpG* cytosine-phosphate-guanine. *DNA* deoxyribonucleic acid

At the molecular level, tobacco smoke alters DNA methylation levels in lung cells and circulating leukocytes [22, 23]. Smoking-related oxidative stress generates DNA demethylation, and nicotine downregulates DNA methyltransferase enzymes that transfer methyl groups to cytosine-phosphate-guanine (CpG) sites [24, 25]. Accordingly, prior epidemiological studies have demonstrated marked differences in total DNA methylation among smokers compared to never smokers [26]. Changes in DNA methylation in gene-coding sequences can alter gene expression and may link tobacco smoke exposure to smoking-related diseases [22]. For example, tobacco smoke has been shown in mouse models to increase methylation of the Bcl-2 promoter [27••]. Bcl-2 promoter methylation lowers Bcl-2 expression, which leads to apoptosis of cells in the alveolar walls and generates premature emphysema [27••]. Similarly, human studies have shown that smoking reduces DNA methylation at the aryl hydrocarbon receptor repressor (AHRR) gene in blood and lung tissue [28–30]. Smoking-induced reductions in AHRR methylation inhibit expression of detoxifying enzymes that remove harmful environmental chemicals including hydrocarbons contained in tobacco smoke [31]. Thus, smoking-induced AHRR demethylation may represent a mechanism of premature lung disease in smokers [32].

Recent studies have shown that DNA methylation levels are also a robust biomarker of biological aging [33]. The “epigenetic clock” effectively predicts biological age in all tissues based upon DNA methylation levels at a collection of CpG sites [34]. The epigenetic clock also quantifies age acceleration, which is a metric of premature aging defined as the difference between the chronological age and the biological or DNA methylation age. Tobacco smoking has been shown in human studies to accelerate DNA methylation age in airway cells and lung tissue, likely by modulating expression of genes that regulate cellular senescence and cell survival [35–38] Accordingly, in two independent population-based studies, accelerated epigenetic aging was associated with incident COPD, which is characterized by premature pulmonary senescence [39, 40]. Specifically, the odds of incident COPD increased by 1–2% per 5-year elevation in epigenetic aging, suggesting that accelerated epigenetic aging is an independent risk factor for age-related lung diseases.

Closely tied to age- and smoking-related epigenetic changes are alterations in cellular protein homeostasis. In natural aging, abnormal proteostasis leads to the accumulation of toxic misfolded protein aggregates [41]. In vitro studies have shown that tobacco smoking also precipitates impaired protein folding in the endoplasmic reticulum (ER) of human lung cells [42•]. The ER regulates protein folding and degradation and activates a stress response when misfolded proteins accumulate in the ER lumen. Smoking additionally inhibits lysosomal-mediated degradation of dysfunctional proteins, which allows abnormal proteins to accumulate in the perinuclear space [43]. Accumulation of dysfunctional proteins generates lung inflammation and dilation of the airways, which are principal components of premature emphysema [44].

In addition to stimulating inflammation in the lung, animal models have demonstrated that smoking-induced protein misfolding inhibits production of functional surfactant proteins [42•]. Pulmonary surfactant is a lipoprotein fluid secreted by type II alveolar cells that decreases surface tension in the alveoli and prevents lung collapse during normal breathing [45]. Smoking-induced inhibition of surfactant protein production induces apoptosis of airway epithelial cells [42]. While epithelial damage typically triggers airway repair mechanisms, tobacco smoke also disrupts airway progenitor cells, impeding alveolar re-epithelialization after injury [46]. Persistent epithelial damage generates alveolar wall destruction and pathological airway remodeling, which are integral components of premature lung aging [47].

Prior studies have shown that tobacco smoking induces premature cellular senescence in lung epithelial cells and basal progenitor cells [15, 48]. Smoking reduces telomere length in both cell populations in vitro, leading to cell cycle arrest and cellular dysfunction [49]. As a result, disordered cell differentiation and impaired epithelial remodeling limit effective cellular repair mechanisms. Tobacco smoke also induces cellular senescence in lung fibroblasts, which are critical for maintaining normal lung architecture [50]. Inhibited wound healing generates architectural disturbances in the alveoli and airway epithelium, which precipitates premature lung disease.

In addition to inducing cellular senescence in airway fibroblasts and progenitor cells, tobacco smoking stimulates an airway epithelial-mesenchymal transition [51]. Human studies comparing smokers to non-smokers demonstrated that some airway epithelial cells undergo biochemical transformations and adopt a mesenchymal cell phenotype following smoking-induced epithelial damage [52]. Transformed mesenchymal cells secrete components of the extracellular matrix and contribute to lung regeneration and healing. However, in the setting of prolonged inflammation and recurrent smoking-related injury, the transformed cells produce a disorganized extracellular matrix and generate fibrosis [53]. Interstitial fibrosis can lead to IPF, which is an irreversible aging-associated lung disease [40].

While a subset of epithelial cells adopt a mesenchymal cell phenotype in response to tobacco smoke exposure, other epithelial cells transform into mucus-secreting cells [54••]. In vitro studies have shown that smoking-related inflammation alters energy production and protein translation in exposed epithelial cells, inducing widespread mucus cell metaplasia. The resulting increase in mucus production contributes to airflow obstruction and premature functional impairment and is a defining feature of many inflammatory lung diseases [55].

Mucus hypersecretion facilitates bacterial colonization of the airways, which leads to recurrent airway infections when coupled with the immunosuppressing effect of chronic smoke exposure [56]. Tobacco smoking suppresses macrophage phagocytosis of bacteria in the lungs and impairs maturation of pulmonary dendritic cells, which are critical activators of the adaptive immune system [57, 58]. Tobacco smoke also inhibits B and T cell immune responses and is associated with decreased immunoglobulin production in animal models [59•]. Dysregulated immunity in the lung can lead to repeated and prolonged respiratory tract infections, which can induce structural changes in the lung and generate premature lung aging [60].

### Particulate Matter

Particulate matter (PM) is a mixture of aerosolized microscopic particles that can be inhaled into the lungs [61]. PM is primarily generated by emissions from motor vehicles and industrial facilities, and fine PM (diameter < 2.5 μm [PM_2.5_]) is small enough to traverse the tracheobronchial tree and deposit in the small airways [62]. PM_2.5_ contains transition metals and organic aerosols that generate ROS and inhibit antioxidant enzyme activity in the lung [63]. As a result, oxidative stress builds in the respiratory tract and accelerates lung aging through multiple pathways (Table 2) [64].

**Table 2:** Summary of particulate matter–induced changes that accelerate lung aging

Reference	Age-related change	Summary	Physiologic effect associated with age-related change
Mostavi et al. [65]	Epigenetic instability	Particulate matter (PM) alters DNA methylation at thousands of CpG sites in the lungs and circulating leukocytes	PM-related changes in DNA methylation upregulate expression of pro-inflammatory cytokines, which trigger architectural changes in the lung
Wang et al. [69]	Inflammation	PM generates a pro-inflammatory milieu that stimulates infiltration of neutrophils and macrophages in the lung	Infiltrating inflammatory cells release proteases that degrade the airway epithelial barrier [71]
Lakey et al. [140]	Oxidative stress	PM contains transition metals and organic aerosols that produce reactive oxygen species in the lung	Oxidative stress damages proteins that form tight junctions in the epithelial cell barrier, diminishing lung epithelial barrier function [78]
Prahalad et al. [75]	DNA damage	PM-induced reactive oxygen species generate DNA strand breaks and suppress DNA repair enzymes in lung epithelial cells	DNA damage stimulates mitochondrial dysfunction in airway epithelial cells, which can induce premature cell death [77]
Chang-Chien et al. [82••]	Telomere shortening	PM exposure decreases expression of human telomerase reverse transcriptase in lung epithelial cells, leading to cell cycle arrest	Unrepaired epithelial injury stimulates proliferation of lung fibroblasts, which produce aberrant extracellular matrix and premature pulmonary fibrosis [86]

Abbreviations: *CpG* cytosine-phosphate-guanine. *DNA* deoxyribonucleic acid. *PM* particulate matter

PM-induced oxidative stress modulates enzymes that regulate DNA methylation, leading to differential methylation at thousands of CpG sites in the lungs and circulating leukocytes [65, 66]. DNA methylation regulates gene expression and may provide a link between particulate air pollution exposure and premature lung aging [67••]. For example, PM_2.5_ exposure has been shown in population-based studies to alter DNA methylation in the interleukin-6 (IL-6) and tissue factor (F3) genes, suggesting PM_2.5_-induced changes in DNA methylation upregulate expression of pro-inflammatory cytokines and acute phase reactants [68]. A corresponding mediation analysis suggested gene-specific methylation mediated the relationship between air pollution and inflammatory biomarkers in plasma. Accordingly, in vitro studies have shown that PM_2.5_ exposure triggers increased epithelial cell production of inflammatory cytokines (IL-6, IL-1β, tumor necrosis factor alpha [TNF-α]) and chemotactic molecules (IL-8, monocyte chemoattractant protein 1 [MCP1]). The resulting pro-inflammatory milieu triggers architectural changes that are characteristic of premature lung aging. PM_2.5_-induced upregulation of IL-1β stimulates mucus hypersecretion in the airway epithelial cells, which generates airflow obstruction [69]. Pro-inflammatory cytokines also stimulate infiltration of neutrophils and macrophages in the lung, which release proteases that degrade the airway epithelial barrier [70, 71]. PM_2.5_ exposure has also been independently associated with AHRR demethylation, [72] suggesting that PM and tobacco smoke may have shared mechanisms of DNA demethylation and may accelerate lung aging through similar biological pathways [73].

The airway epithelial barrier provides both physical and immunological protection against inhaled foreign antigens, and damage to the barrier drives pathogenesis of age-related lung diseases [74]. In addition to generating inflammatory injury to the epithelial barrier, PM_2.5_-induced ROS generate DNA strand breaks and suppress DNA repair enzymes in airway epithelial cells [75, 76]. DNA damage stimulates mitochondrial dysfunction, which can induce epithelial cell death [77]. In vitro studies have demonstrated that oxidative stress also damages the proteins that form the tight junctions in the epithelial cell barrier, thereby diminishing the barrier function of the airway epithelial layer [78, 79]. In response, alveolar progenitor cells are activated and recruited to repair the injured alveolar barrier [80]. However, when epithelial repair mechanisms are impaired, pathological airway remodeling ensues.

Prior in vitro studies have shown that inhaled PM decreases the viability of epithelial progenitor cells. PM_2.5_ exposure decreases expression of human telomerase reverse transcriptase in lung epithelial cells [81]. As a result, epithelial cell telomeres are shortened and cell cycle arrest ensues [82••]. Cellular senescence in type II alveolar epithelial cells limits the regenerative capacity of the lung epithelium, leading to impaired wound healing and increased inflammation [83]. Unrepaired epithelial injury also induces proliferation of lung fibroblasts [84, 85]. Activated fibroblasts increase collagen deposition, and excessive production of extracellular matrix leads to aberrant alveolar remodeling and fibrosis [86]. Accordingly, epidemiological studies have shown that PM exposure is a known risk factor for IPF, which is a progressive age-related lung disease [87].

### Ground-Level Ozone

Tropospheric ozone forms when emissions from industrial plants and motor vehicles chemically react in the presence of UV light [88]. Ozone is not filtered by the upper airways, which allows inhaled ozone to deposit in the lower respiratory tract [89]. When ozone comes into contact with lung epithelial cells in vitro, it alters expression of tight junction proteins in the epithelial barrier [90••]. Disintegration of tight junctions increases permeability of the epithelial barrier, which stimulates release of inflammatory cytokines and ROS [91]. In turn, ozone-induced ROS generate mitochondrial dysfunction [92•]. Acute ozone exposure diminishes mitochondrial energy storage and decreases mitochondrial oxygen consumption in the lung, leading to release of mitochondrial ROS. Mitochondrial ROS activate the NLRP3 inflammasome, which is a protein complex that induces inflammation-mediated cell death and generates alveolar wall destruction [93]. Accordingly, in prior epidemiological studies, chronic ozone exposure was associated with lung function impairment and emphysema independent of smoking [94••]. Long-term ozone exposure was also associated with shortness of breath and impaired functional status resulting from respiratory symptoms, suggesting ambient air pollution may contribute significantly to respiratory symptoms that characterize premature lung aging.

### Sulfur Dioxide

Fossil fuel combustion generates sulfur dioxide, which is a toxic gas that contributes heavily to air pollution in industrialized countries [95]. Human studies have demonstrated that sulfur dioxide converts to sulfuric acid after inhalation and increases bronchial reactivity and bronchoconstriction, which are hallmarks of reactive airway disease and asthma [96]. Sulfur dioxide also decreases mucociliary clearance, which increases the viscosity of airway mucus and promotes pathogen colonization and reproduction [97]. Pathogen colonization in the airways promotes infiltration by immune and inflammatory cells, which in turn promotes airway remodeling and premature lung aging [98].

### Nitrogen Dioxide

Fossil fuel combustion generates nitrogen dioxide, which is a primary source of urban air pollution [99]. Nitrogen dioxide is a water-soluble gas that deposits in the small airways where it is converted to nitrous and nitric acids [100••]. Nitric acids directly damage airway epithelial cells, leading to a chemical pneumonitis that manifests as pulmonary edema [101]. Nitrogen dioxide exposure also suppresses alveolar macrophage-mediated production of inflammatory cytokines in response to bacterial infection, which dampens the immune response. The pollutant further diminishes respiratory immunity by reducing mucociliary clearance in the lower respiratory tract of animal models, leading to impaired clearance of respiratory pathogens [102]. Immune dysregulation in the lungs increases susceptibility to respiratory infections, which can lead to cellular and structural damage in the airways [103].

### Silica Dust

Silica is a common mineral that is a large component of granite and sandstone rocks [104]. Silica exposure can occur after any activity that requires breaking ground or handling silica-containing stone. Inhaled crystalline silica particles deposit in the distal airways of the lungs and are phagocytosed by resident macrophages, which release ROS and inflammatory cytokines [105]. Animal models have demonstrated that silica-induced ROS disrupt a telomere-binding protein complex that preserves telomere length in progenitor lung cells [106]. The resulting telomere attrition generates DNA damage in type II alveolar epithelial cells, leading to cellular senescence and apoptosis. Increased alveolar cell loss triggers aberrant healing mechanisms in lung fibroblasts, which proliferate and increase collagen production around silica particles [107]. The resulting interstitial fibrosis leads to decreased lung compliance and impaired gas exchange, which are hallmarks of premature lung aging.

### Coal Dust

Coal mining, transport, and processing generate airborne respirable dust that can deposit in the small airways of the lungs [108]. Coal dust cannot be eliminated from the lungs but rather is engulfed by macrophages that reside in the alveolar space. Activated macrophages release TNF-α and IL-6 in vitro, which stimulate infiltration of neutrophils and lymphocytes in the lungs. Neutrophils secrete elastases that break down elastic fibers, leading to dilation and destruction of the alveolar walls [109]. Alveolar macrophages also secrete insulin-like growth factor-1 (IGF-1) and platelet-derived growth factor (PDGF), which recruit fibroblasts to sites of coal dust deposition and stimulate fibroblast proliferation [110] Activated fibroblasts upregulate collagen production, leading to collagen accumulation in pneumoconiotic lesions in the lungs. Areas of focal emphysema and pneumoconiotic nodules distort the lung architecture and generate premature lung function impairment.

### Asbestos

Asbestos fibers are durable minerals that were historically used in construction and insulation because of their resilience and affordability [111]. Deteriorating buildings and asbestos-containing products can generate airborne asbestos fibers that deposit in the distal airways [112]. Alveolar macrophages are too small to completely engulf larger asbestos fibers, and incomplete phagocytosis generates biomineralization and formation of iron-rich envelopes around asbestos fibers [111]. Animal models have shown that asbestos-iron complexes trigger mitochondrial ROS production in alveolar epithelial cells, mesothelial cells, and macrophages [113]. Persistent oxidative stress induces mitochondrial DNA damage, triggering apoptosis of mesothelial cells and type II alveolar epithelial cells. Asbestos exposure also activates p53 expression in key progenitor cells, leading to cellular senescence in alveolar epithelial and mesothelial cells [114]. Cellular senescence in progenitor cells impairs physiologic repair mechanisms and triggers an exaggerated fibroblast response characterized by pathological collagen deposition. Proliferating fibroblasts generate interstitial fibrosis, which is a hallmark of asbestosis and premature lung aging [115]. Notably, tobacco smoking hinders clearance of asbestos bodies from the human lung, leading to exaggerated pulmonary toxicity in smokers with asbestos exposures [116].

### Bioaerosols

Indoor air contains ubiquitous biological contaminants including bacteria, viruses, and fungi [117]. Indoor dust also contains bacterial extracellular vesicles (EVs), which are membrane-bound nanoparticles produced from Gram-negative bacteria that can reach and accumulate in the lung alveoli [118]. Inhalation of bacterial EVs activates the innate immune response and triggers production of inflammatory cytokines including TNF-α and IL-6 in vitro [119]. Cytokine release triggers an influx of neutrophils in the lung, which release protease and elastase enzymes that destroy structural components of the alveolar septa [109]. Repeated exposure to bacterial EVs alters the lung architecture by damaging alveolar walls and generating alveolar enlargement, as well as by increasing collagen deposition in the airways. The prolonged inflammation that follows EV exposure can generate mucus gland hyperplasia and emphysema and indicates that dust EVs may contribute significantly to premature lung aging [120••].

## Strategies for Improving Environmental and Respiratory Health

**Fig. 2:**
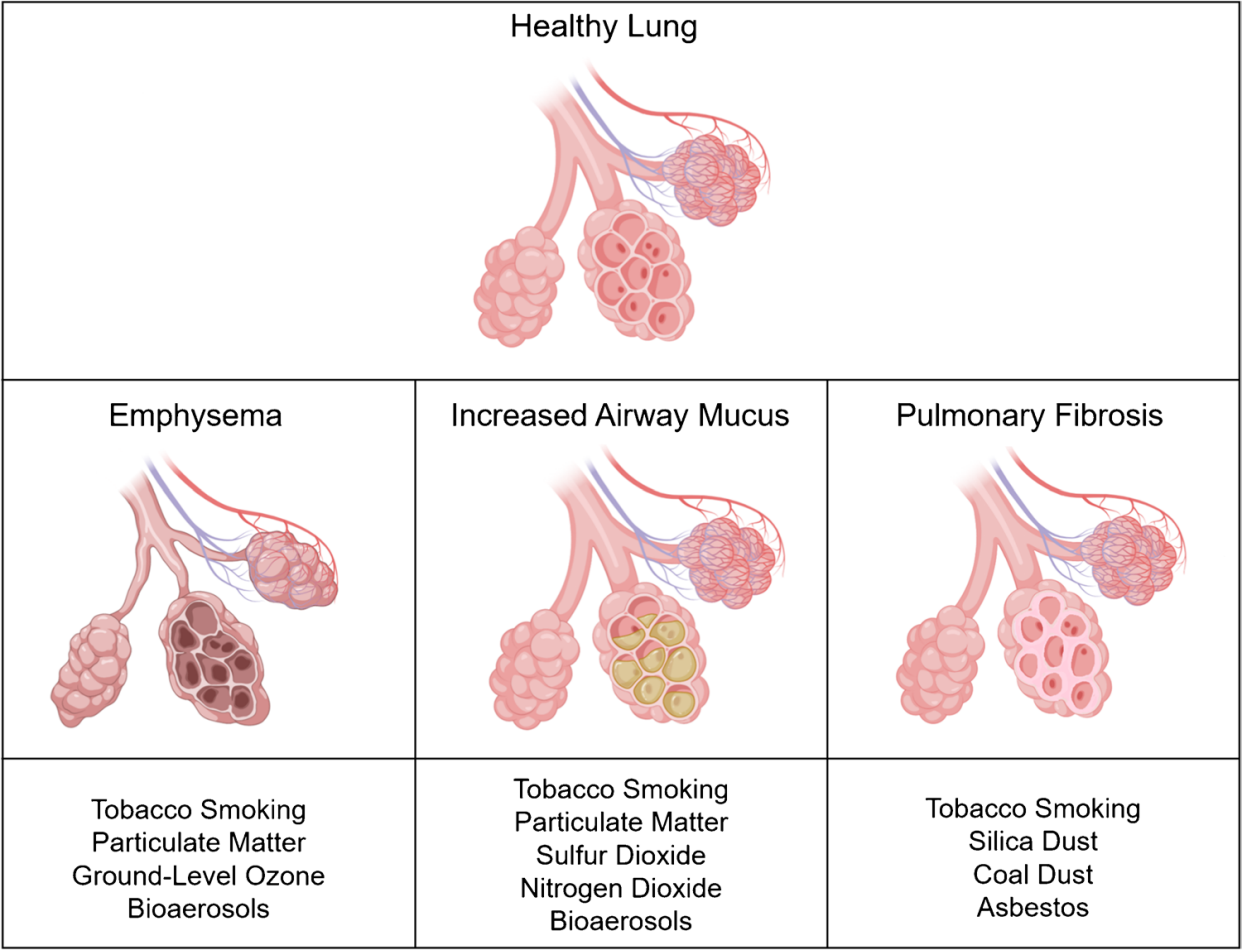
Environmental exposures and associated age-related changes in the lung^142^. Environmental exposures impair cellular repair mechanisms in the lung, leading to structural alterations including emphysema, mucus hypersecretion, and pulmonary fibrosis that generate functional impairment and characterize premature lung aging

Environmental exposures accelerate lung aging and contribute to the development of age-related respiratory diseases (Figure 2). Inhaled antigens generate oxidative stress and inflammation that lead to destruction and fibrosis of the lung parenchyma, which are irreversible distortions of the lung architecture [121]. Thus, evaluation and minimization of noxious environmental exposures are critical to improve lung health and prevent age-related lung diseases.

Several strategies can help reduce exposure to ambient air pollution. Portable or centralized air filters reduce concentrations of indoor air pollutants, which reflect a combination of pollutants from cooking and organic dust as well as outdoor source pollutants that infiltrate indoors [122, 123]. Clinical trials have shown that high efficiency particulate air (HEPA) in-duct filtration systems reduce indoor particulate concentrations and improve microvascular health, which is a determinant of lung health [124, 125]. Similarly, individuals who commute via personal vehicles or public transportation can reduce in-vehicle particulate exposure through use of cabin filters and air conditioners to stimulate air recirculation [126].

Avoiding rigorous outdoor activity when air pollutant concentrations are high can also help minimize harmful exposures. Air pollution monitoring networks such as the Environmental Protection Agency’s Air Quality Index measure concentrations of harmful air pollutants and forecast air pollutant levels using mathematical models [127]. When air pollution levels are elevated, minimizing outdoor activity and reducing rigorous outdoor exercise can decrease inhalation of harmful pollutants [128, 129]. Reducing exposure to high-pollution microenvironments can also reduce noxious exposures. For example, avoiding physical activity near high-traffic roads can help optimize respiratory health [130]. Similarly, smoking cessation and complete removal of secondhand smoke exposure are essential to preserve lung health among smokers and their family members. There are multiple proven interventions to aid with smoking cessation and removal of residual smoke pollution [131].

With regard to occupational exposures including silica dust, coal dust, and asbestos, minimizing dust exposure is the most effective prevention strategy. While engineering controls including dust extraction systems are the primary method for reducing exposures, individual prevention strategies including wearing a mask and removing dust from skin and clothing can help reduce harmful exposures [132]. Secondary prevention strategies including monitoring for lung function impairment and early radiographic signs of disease in high-risk individuals can help prevent severe disease, as miners with radiographic evidence of lung disease are legally entitled to work with enhanced protections against dust exposure [133].

In addition to minimizing inhaled environmental exposures, primary prevention strategies in the form of influenza, pneumococcal, and Covid-19 vaccinations can minimize risk of respiratory infections and promote lung health among individuals with high-risk environmental exposures [134]. Individuals over the age of 65, and particularly those with chronic medical conditions, are recommended to stay up to date on vaccinations in order to prevent severe respiratory infections.

Prior research has suggested that pharmacological and dietary interventions may reduce susceptibility to inhaled exposures. An epidemiological study showed that nonsteroidal anti-inflammatory drugs (NSAIDs) partially attenuated lung function decline after PM exposure, [135] and a small trial demonstrated that NSAID use attenuated lung function decline in adults exposed to ozone [136]. Similarly, B vitamin supplementation prevented PM_2.5_-induced alterations in DNA methylation levels in a small trial of adults, suggesting B vitamins can neutralize PM-related oxidative stress [137]. Dietary antioxidants including vitamin C also preserved lung function in a study of young adults exposed to ozone [138]. Finally, stem cell therapies are emerging as potential mitigators of environmental pollutants, and transplantation of adipose-derived stem cells attenuated PM2.5-induced lung inflammation in mice [139]. However, while preliminary studies are promising, further research is required before pharmaceutical, biological, and lifestyle interventions can be safely recommended to minimize or reverse damage from inhaled environmental exposures.

## Conclusions

The lungs are one of few organs in the body that continuously interface with the external environment. Environmental exposures trigger oxidative and inflammatory stress that damage the lung parenchyma, impair physiologic repair mechanisms, and induce accelerated lung aging. Premature lung aging manifests as lung function impairment and overt lung disease and causes significant global morbidity and mortality. While individual interventions that minimize noxious environmental exposures and reduce risk of respiratory infections can optimize lung health, cohesive national and international policies that minimize carbon emissions are required to decrease the global burden of inhaled environmental exposures. In addition, novel research is required to expand our knowledge of therapeutic interventions that may slow or prevent premature lung aging. Future research in the following areas may expand available strategies to prevent age-related lung diseases:

- Human trials examining pharmacologic interventions (i.e., NSAIDs) are required to determine if medical interventions protect the lungs from environmental pollutants.
- Larger human trials are required to determine whether dietary interventions including antioxidant supplements (i.e., vitamins B, C, and E) can mitigate the impact of inhaled exposures on lung health.
- While stem cell therapies represent an emerging and promising field, additional studies in animal models and eventually humans are required to validate the effectiveness of stem cells in preserving lung health.
